# Investigating people’s metacognitive insight into their own face abilities

**DOI:** 10.1177/17470218231218662

**Published:** 2023-12-16

**Authors:** Robin SS Kramer, Jeremy J Tree

**Affiliations:** 1 School of Psychology, University of Lincoln, Lincoln, UK; 2 Department of Psychology, Swansea University, Swansea, UK

**Keywords:** Metacognition, insight, face recognition, face matching, self-report

## Abstract

Within the domain of face processing, researchers have been interested in quantifying the relationship between objective (i.e., performance on laboratory tests of recognition and matching) and subjective measures of ability (typically, self-report questionnaires). Put simply, do people show high levels of metacognitive insight into their own abilities with faces? Although several studies have suggested that the association between these two types of measures may only be moderate, there remain several important issues that require consideration before this question can be sensibly investigated. First, specificity is needed regarding both objective and subjective measurements because both tend to span a wide range of potentially separable abilities. Second, experimental tasks appear to focus on different contexts to those tapped in self-report questionnaire items. Third, recent issues with statistical approaches and visualisation can result in numerical artefacts and misinterpretations. Finally, the sizes of population-level insights suggested by recent work provide only limited information regarding individuals within these populations, and so researchers aiming to identify people at the extremes of ability must be careful when drawing conclusions. Taken together, we argue that more attention to these issues is needed when attempting to investigate metacognitive insight within this domain.

Researchers have established the existence of substantial individual differences in face recognition and matching abilities (e.g., Burton et al., 2010; Duchaine & Nakayama, 2006). Those who demonstrate exceptionally good performance are often referred to as “super-recognisers” (e.g., Bobak, Hancock, et al., 2016; Russell et al., 2009), while those who perform exceptionally poorly have been termed “face blind” or prosopagnosic (e.g., Bate et al., 2019; Dalrymple & Palermo, 2016; White et al., 2017). While a variety of experimental tasks have been devised with the aim of identifying individuals at these two extremes (e.g., Bate et al., 2018), the possibility of a simple route to identification has also been investigated through self-report.

Through exploring the association with self-report measures, researchers have uncovered only small-to-moderate correlations with tests of face memory (e.g., Bobak et al., 2019; Gray et al., 2017; Matsuyoshi & Watanabe, 2021; Palermo et al., 2017) and matching (e.g., Shah, Sowden, et al., 2015). As a result, the question of whether people have insight into their abilities with faces (referred to as *metacognition*) has been the topic of much debate (e.g., Bindemann et al., 2014; Grüter et al., 2011; Tree, 2011; Tree & Wilkie, 2010). However, before proceeding, a distinction should also be highlighted between the *type* of metacognitive awareness under scrutiny—an issue often overlooked. Here, we focus specifically on *global* or task-level metacognitive awareness. That is, measures representing overall estimates of subjective and objective face ability (e.g., scores on face matching tests or self-report questionnaires). These are typically used in the identification of high or low performers, and for the consideration of self-insight within the population, and so may likely have more functional/clinical relevance (e.g., Seow et al., 2021).

However, several researchers have begun to investigate more *local*, trial-level metacognitive awareness (e.g., Ji & Hayward, 2021). By analysing the level of confidence assigned to individual responses on a specific task, for instance, we know that people who score higher on tests of face matching are also better able to differentiate between correct and incorrect responses, showing greater confidence in the former (e.g., Kramer, 2023; Kramer et al., 2022). Again, for the sake of brevity, we would seek to stress that the “framing” of what is being measured as metacognitive awareness may have important implications for subsequent interpretation. It appears to be an open question whether these different levels of focus (i.e., local vs global) are indeed measuring entirely related or somewhat separate abilities, and thus again it is likely important in future to avoid conflating findings that may be measuring very different things.

In any case, the potential for using self-report as a tool for “diagnosing” any kind of sub-group of a population who may show extreme performance (at either end of the distribution) has obvious utility (e.g., to identify and provide the appropriate resources), and so unsurprisingly, there has been an increasing focus on establishing appropriate tools to achieve this goal (e.g., Bate et al., 2018). At the same time, the example of diagnosing prosopagnosic presentation can act as an interesting test case for this endeavour, while the implications of this work could potentially generalise to several other areas, including the diagnosis of early-stage dementias like Alzheimer’s disease (Jenkins et al., 2015, 2021). In such domains, people may report that their memories are deteriorating and yet cognitive assessments signal “normal” performance. As a result, they will be left to experience no diagnosis for a long time—a poor outcome for both themselves and their families. Therefore, research activities focussed on cognitive performance and insight may have quite broad implications beyond the specific topic of face processing discussed here.

With the goal of investigating insight into one’s ability with faces, much previous research and debate has typically focussed on the strength of association (i.e., metacognitive *sensitivity*) between *objective* and *subjective* measures of the domain in question, and this will be the main focus of our subsequent discussion. However, metacognitive *bias* (often calculated as the difference between self-reported and objective measures) also relates to this issue, and we would highlight that emerging work suggests it is a fruitful avenue of research for a more complete understanding of metacognitive processes in this context (e.g., DeGutis et al., 2023). Nonetheless, from the outset, the decision of how to first operationalise each of these subjective/objective measures poses a substantial problem for researchers and is a major obstacle to reaching any clarity in this field. In the following sections, we summarise the issues faced when considering each type of measure, the possibility of determining the associations between them, and what such associations may or may not imply.

## How best to measure “objective” face ability?

Before considering insight, we first need to measure how well someone actually performs on the objective face task in question. Here, it becomes clear that face processing can encompass a great number of different day-to-day examples, including (a) *identification* (“that’s my friend Bob”), (b) *recognition* (“I’ve seen that man before somewhere”), and (c) *matching* (“this passport photo matches the woman in front of me”). Our focus will be largely on the latter two examples as these are most often investigated by laboratory tests and are strictly face-focussed, but it is perhaps ironic to note that the first is, we suspect, what the general public might consider to be a cardinal example of day-to-day “face ability.”

In terms of laboratory tests of face memory and recognition, the Cambridge Face Memory Test (CFMT; Duchaine & Nakayama, 2006; Russell et al., 2009), for instance, involves the learning of unfamiliar faces and their subsequent recognition from line-ups containing a previously seen or novel image of the face (sometimes with noise applied). Faces are learnt over a 3-s period via three images of an identity taken at different viewing angles, and recognition is tested within seconds/minutes of this learning. Researchers have also considered the recognition of familiar faces. Such famous face tests typically depict celebrities before they became well-known (e.g., the Before They Were Famous Test, BTWF; Russell et al., 2009) to avoid ceiling effects. However, these familiar and unfamiliar (newly learnt) face tests are often imperfectly correlated. For example, the association between CFMT and BTWF scores appears to be moderate to large (e.g., .33—McCaffery et al., 2018; .70—Russell et al., 2009; .70—Wilmer et al., 2010), suggesting that the two tasks represent different but related abilities. In general, evidence suggests that familiar and unfamiliar faces are processed in qualitatively different ways (see Burton, 2013, for a discussion of this), and thus task selection for subsequent correlation with subjective measures of face ability may have important implications.

In contrast, tests of face perception remove the need for any learning or memory. The Cambridge Face Perception Test (CFPT; Duchaine et al., 2007), for instance, involves showing a target face, along with six morphs, i.e., blends of the target face with one of six other identities, varying in the proportion contributed by the target itself. Viewers must arrange the morphs in order of this target face proportion (simply, their similarity to the target). In addition, researchers employ tests of face matching, requiring viewers to decide whether two images depict the same person or not (e.g., the Glasgow Face Matching Test [GFMT]—Burton et al., 2010; the Kent Face Matching Test [KFMT]—Fysh & Bindemann, 2018), or which line-up image also depicts the target identity (e.g., the Benton Facial Recognition Test; Benton & Van Allen, 1968; Murray et al., 2022). As noted, all information is presented simultaneously and no memory is required. Importantly, even within this category of tests, performance correlations are imperfect (e.g., −.34 between KFMT and CFPT scores; Fysh & Bindemann, 2018). In fact, the association between two face matching tests may only be moderate (e.g., .45 between KFMT and GFMT scores; Fysh & Bindemann, 2018). In addition, others have argued that face matching tasks may well also be tapping two separable abilities: (1) realising that two images show the same person; and (2) realising that two images show two different people. Indeed, scores for these two types of trials demonstrate only a small correlation (e.g., .11—Megreya & Burton, 2007). As a consequence, it appears that not only do different iterations of matching task correlate to a variable extent, but *within* the task itself, different skills may be being used. Again, any approach that simply chooses to correlate a given matching task with global, subjective judgements of “face ability” will mask these underlying complexities for the objective measure selected.

Given that different tasks within each of the two categories above show imperfect associations, it comes as no surprise that correlating scores between categories of tests produces a similarly imperfect result. For instance, CFMT scores correlate only moderately with GFMT (e.g., .45—McCaffery et al., 2018) and KFMT scores (e.g., .29—Fysh & Bindemann, 2018), again having consequences for the wider “insight” research question. Put simply, if the aim is to determine how much insight an individual has regarding their own face abilities, it is clearly important to reflect on the means by which we measure objective face ability—namely, key decisions about specific domains (matching vs recognition) and the stimuli under consideration (e.g., unfamiliar/novel faces vs familiar faces)—particularly as it is clear that the tasks available have a number of flaws, i.e., poor intercorrelations despite putatively measuring the same thing. As a consequence, perhaps it is advisable when investigating the question of insight into face ability to preface this with “what face ability do you mean?” or even “what aspect of this particular face ability do you mean?”

## How best to measure “subjective” face ability?

To assess subjective ability with faces, researchers have typically used self-report measures. At their simplest, these have comprised a single question/item (e.g., Bindemann et al., 2014; Bobak, Pampoulov, et al., 2016; Bowles et al., 2009; Gauthier et al., 2014; McGugin et al., 2012; Rotshtein et al., 2007). However, single items are likely to be subject to noise in their measurement, and any biases due to participant self-esteem, demand characteristics, and so on. Admittedly, these item–performance associations are not always much worse than those resulting from multiple-item questionnaires, e.g., a correlation of .30 between CFMT + scores and a single rating of one’s general face recognition ability (Bobak, Pampoulov, et al., 2016).

In any case, several multiple-item questionnaires have been developed with the aim of better measuring self-reported ability with faces. These include the Stirling Face Recognition Scale (SFRS; Bobak et al., 2019), the 20-item Prosopagnosia Index (PI20; Shah, Gaule, et al., 2015), the Cambridge Face Memory Questionnaire (CFMQ; Arizpe et al., 2019), the Hong Kong Prosopagnosia Questionnaire (Kennerknecht et al., 2008), and its shortened 11-item version (HK11; Matsuyoshi & Watanabe, 2021), and scores across these tests are strongly correlated (e.g., .82 for the PI20 and HK11; Matsuyoshi & Watanabe, 2021). This is perhaps unsurprising given that questions across measures are often highly similar—e.g., “I find it easy to picture individual faces in my mind” (PI20) and “I can easily form pictures of close friends in my mind” (HK11).

Critically, this multiple-item approach has resulted in questionnaires that encompass a broad range of “face processing” activities, including recognising characters in films, mentally picturing faces, reading facial expressions, noticing family resemblance, and so on—hence the global focus we highlighted above. Since the objective tests discussed earlier likely tap different aspects of face ability, the association between any given task and a composite score from a broad, global self-report measure may be problematic. How can we subsequently determine whether people have accurate insight when it is unclear as to which ability we are addressing? Indeed, analysis of the SFRS (Bobak et al., 2019), for example, revealed two relatively distinct components related to face processing and, somewhat separately, face memory.

Moreover, attempting to cover a variety of seemingly different contexts within these self-report questionnaires, the items themselves seem to vary in the types of metacognition involved. Considering examples from the PI20, for instance, we see that many pertain to how individuals think or feel about themselves (e.g., “I am very confident in my ability to recognize myself in photographs”), but some items require people to predict what others are thinking (e.g., “My friends and family think I have bad face recognition or bad face memory”) or to compare themselves to others (e.g., “My face recognition ability is worse than most people”). As such, these questionnaires again appear to encompass a wide range of items, potentially measuring many different skills and metacognitive processes, suggesting a great deal of heterogeneity rather than a single concept.

Finally, we highlight that, separate from considering the content of the questionnaires themselves, there may be biases in the use of such self-report measures. For example, men were found to under-report prosopagnosia symptoms relative to women (Murray & Bate, 2019). Similarly, older adults reported fewer face recognition issues while simultaneously declining in their actual performance (DeGutis et al., 2023). Such demographic biases represent further difficulties when measuring subjective abilities that must be included in any interpretations of associations.

## Internal reliability

If researchers plan to determine the association between self-reported and actual abilities, it is necessary to first consider the *reliability* of the two measures in question. Regarding the internal reliability/consistency of a measure, researchers must evaluate whether all of the items are measuring the same construct. If this is not the case, then the maximum expected correlation between two tasks, or a task and a questionnaire, will be notably less than one (Bobak et al., 2023).

Subjective measures of ability show acceptable levels of reliability as measured by Cronbach’s α (e.g., .96 for the PI20—Shah, Gaule, et al., 2015; .85 for the HK11—Matsuyoshi & Watanabe, 2021). It is far less common that researchers have reported the internal reliability of face perception tasks. Where available, these were typically lower than for subjective measures (e.g., .89 for the CFMT, .74 for the CFPT_upright_, and .50 for the CFPT_inverted_—Bowles et al., 2009; .67 for the GFMT and .60 for the KFMT—Bobak et al., 2023). Given such values, it is important to understand that the maximum expected correlation between the HK11 and the KFMT, for example, would be √(.85 × .60) = .71 (Verhallen et al., 2017). Clearly, this has repercussions for both the measurement and demonstration of metacognition within a given study, and highlights again the issue of improving the particular measures we might deploy.

Moreover, one must remember that a high Cronbach’s α does not necessarily mean high reliability because the value is increased simply through the inclusion of more trials or items (e.g., Streiner, 2003). It is also worth noting that alpha is a property of the scores calculated for a specific sample and so it is recommended that researchers do not rely on previously published estimates and instead measure Cronbach’s α each time the test or questionnaire is administered (Streiner, 2003).

## Do objective and subjective measures align with each other and with “real-world” performance?

To appropriately understand the degree of “insight” individuals have into their face-based abilities, it is clearly important to consider the alignment between self-report questionnaires and experimental tests of face abilities. As we have hinted at earlier, if these are not tapping into the same skills then it becomes unclear as to what any association would mean in terms of metacognitive insight. Laboratory tests of recognition typically involve learning unfamiliar faces over a few seconds, with recognition tested moments later, while perceptual/matching tasks present images on screen simultaneously and therefore do not require any memory or learning. In contrast, self-report questionnaires, as we have seen, are mostly composed of items designed to measure face recognition in everyday situations. For example, items include “at family gatherings, I sometimes confuse individual family members” (PI20), “I do not recognize people the day after a brief meeting” (HKPQ), and “I can recognize casual acquaintances out of context” (CFMQ). The issue, therefore, is that these items may have little overlap with the abilities measured in the above-mentioned tests. It is even less clear as to whether such items relate to the CFPT and the variety of face matching tests currently available. Although Kramer (2021) found that face recognition performance based on learning identities via 30 s videos showed only a moderate correlation (.34) with self-reported ability with the HK11, it may be that faces learned using more naturalistic paradigms (e.g., Kramer et al., 2017) result in stronger associations with these self-report measures.

Interestingly, a number of items in these questionnaires refer specifically to the recognition of famous faces. For example, “I sometimes find movies hard to follow because of difficulties recognizing characters” (PI20), “I recognize famous people immediately” (HKPQ), and “I can recognize well known actors/actresses when watching a movie” (CFMQ). Perhaps a subset of items like these may well be able to better tap specific skills like those tested with the BTWF, although a single-item self-report measure designed to overlap with this test’s specific requirements showed only a moderate association (.42) with actual test scores (McCaffery et al., 2018).

Of course, while the alignment of objective and subjective measures is crucial for quantifying the insight that individuals have into their own face processing abilities, this does not speak to how well both of these measures align with real-world challenges. If the idea is to identify those with excellent face abilities with the aim of selection/recruitment to particular security roles, for instance, then more consideration of this question is vital (Devue, 2019; Ramon et al., 2019). Indeed, even the identification of “good enough” individuals (e.g., police officers) when considering performance on face matching is problematic, for instance, if tests fail to align with real-world contexts but also with each other (e.g., the GFMT and KFMT, which are often used interchangeably but show only moderate correlations with each other).

## How should we visualise the association?

To investigate the relationship between self-reported and actual abilities with face perception, perhaps the most intuitive approach is to consider the simple (y) versus (x) scatterplot with a line of best fit (e.g., Gray et al., 2017). Relationships illustrated in this way are clear to interpret, with the added benefit that simulations of what we might expect from random noise are easily identified (Nuhfer et al., 2016). Unfortunately, a common convention within the metacognition literature is to use *performance quartiles* (Kruger & Dunning, 1999; see Zhou & Jenkins, 2020, for a face perception example). By dividing participants into quartiles based on their actual abilities, and then plotting these against their self-reported abilities, researchers invariably produce a pattern often interpreted as the worst performers (lowest quartile) overestimating their performance and the best (highest quartile) underestimating theirs. This graphical convention is especially prone to regression to the mean (e.g., Kramer et al., 2023; Nuhfer et al., 2016) because individuals with the lowest and highest actual scores are less likely to provide self-reported scores that are also at the extremes. Indeed, even shuffled or random data produce this same pattern of results (Kramer et al., 2022). We would therefore strongly advise face perception researchers to avoid such an approach when investigating insight into one’s face abilities.

Another approach to visualisation is to display data as a (y-x) versus (x) scatterplot, where the former could be interpreted as metacognitive accuracy in that it represents the difference between self-reported and actual abilities. Unfortunately, random number simulations demonstrate that variables considered in this way will result in an association of −.70 (thus, a numerical artefact). In addition, self-reported and actual abilities are typically bounded (i.e., there will be minimum and maximum values that could be obtained), producing floor and ceiling effects. For instance, individuals who score high on a test would have limited or no potential to overestimate their performance, which is not the case for low scorers. Again, such artefacts can alter the apparent association when depicted in this way.

## What does a “moderate” association even mean?

Associations between self-reported and actual abilities in face perception tend to be labelled as small to moderate in size (following statistical conventions). For example, the PI20 showed correlations of −.39 (Gray et al., 2017) and −.47 (Shah, Sowden, et al., 2015) with scores on the CFMT and GFMT, respectively. Interestingly, a metasynthesis carried out across a variety of non-face domains found a mean correlation of .29 between ability self-evaluations and performance outcomes (Zell & Krizan, 2014). This might suggest that metacognitive awareness in the domain of face processing is comparable with, or perhaps better than, that for other cognitive domains. This is notable, given individuals may be less likely to receive day-to-day feedback regarding their face-related abilities (e.g., if we confuse celebrities on the television, we might not be corrected) in comparison with those involving some academic (e.g., mathematical) or sports-based (e.g., visuomotor) domains. As a consequence, the interpretation of any association may well need to take such issues into account.

Estimates of these associations may also be inflated. When considering the correlation between subjective and objective measures, the composition of the sample is important. While most of the population (“controls”) might only show moderate associations between subjective and objective face perception measures, the inclusion of special populations (e.g., super-recognisers) can artificially inflate these estimates. For example, by incorporating seven super-recognisers, who (by definition) produced high objective scores but were also aware of their exceptional abilities (through previously receiving feedback on their performance), the correlation between CFMT+ and SFRS scores increased from .36 to .50 (Bobak et al., 2019). As such, researchers need to be aware that individuals may have prior knowledge of their abilities (e.g., a prosopagnosia diagnosis) and that this could subsequently influence any conclusions regarding metacognitive measurement. Estudillo and Wong (2021) argued that those individuals who perform at the “extremes” of a population distribution have greater insight than average, although this has subsequently been shown to be an analytical artefact (Kramer et al., 2023).

Even when considering only typical performers, it is important to stress that associations between self-reported and actual abilities represent *population-level* trends. This is particularly relevant as researchers are often interested in identifying *individuals* using self-report measures as a proxy, and this is where issues with interpretation become evident. Let us assume that the association between self-reported and actual face perception abilities is approximately .50 (perhaps a realistic upper limit based on the current literature). Many researchers would interpret this as the minimum value considered to be “large” (Cohen, 1988) and might draw conclusions regarding particular individuals as a result. For example, one might argue that a person is likely to produce a high score on the GFMT based on their high self-reported face recognition skills according to the PI20. Ignoring the various measurement issues we have already highlighted, we would also point out that, statistically speaking, such a statement can be inaccurate and misleading.

Following the approach of Mõttus (2022), we can trisect both measures to form tertiles (i.e., by ordering scores from the lowest to highest and dividing into three equally sized groups), labelling these as “low,” “medium,” and “high” scorers. Assuming the “best-case” correlation of .50, we can state that 49% of people are expected to have a similar trait level (i.e., fall within the same tertile) in both self-reported and actual abilities. More specifically, of those scoring high or low in self-reported ability, 55% would score similarly in actual ability, whereas 31% would have a medium, and 14% an opposite score (see Table 1, adapted from Mõttus, 2022). Among medium scorers in self-reported ability, only 38% would have the same trait level in actual ability. (Note that random chance correspondence is 33%).

**Table 1.** Illustrating how a correlation of .50 between self-reported and actual face perception abilities would translate to the sample’s distribution across tertiles.

Self-reported ability	Actual ability		
	Low	Medium	High
Low	54.87%	31.12%	14.02%
Medium	31.12%	37.77%	31.12%
High	14.02%	31.12%	54.87%

Low, medium, and high tertiles are calculated for self-reported and actual face perception abilities separately. Random chance correspondence is 33.33%.

Extending this approach, lower associations between self-reported and actual face perception abilities would result in distributions even closer to random correspondence. For example, Gray and colleagues’ (2017) correlation of −.39 between PI20 and CFMT scores translates to *only half* of those (49.7%) with a low or high value on one measure having a similar value on the other.

This approach highlights the need for researchers to avoid drawing categorical conclusions about individuals based on population-level statistical trends. If necessary, such statements should be qualified, such as “in a large group of people, higher self-reported face perception abilities tend to be associated with higher actual abilities.” Beyond this, any conclusions are likely to mislead the reader. In addition, the above examples entirely ignore all of the “noise” that is added by the many measurement issues we have raised (e.g., the misalignment of objective and subjective measures).

## Conclusion

We have identified several issues that require further consideration when investigating metacognitive insight into one’s own face abilities. First, objective measurements tend to span a wide range of abilities, with substantial variability (both within and across tasks), and likely refer to different contexts to those tapped in self-report questionnaire items. Second, recent issues with approaches to statistical analysis and visualisation can result in numerical artefacts and subsequent misinterpretations. Finally, it is important to realise that the sizes of population-level insights suggested by recent work provide very limited information regarding *individuals* within these populations, and so researchers must be careful when drawing conclusions based on these results.
